# Supplementary material for: Non-Gaussian noise spectroscopy with a superconducting qubit sensor
Source: Nat Commun. 2019 Sep 16;10:3715. doi: 10.1038/s41467-019-11699-4 (PMC6746758; doi:10.1038/s41467-019-11699-4)
Supplement: Supplementary file 1 — Supplementary Information [file 41467_2019_11699_MOESM1_ESM.pdf]

Supplementary Information: Non-Gaussian noise spectroscopy with a superconducting qubit sensor

Youngkyu Sung[1,2], Félix Beaudoin[3], Leigh M. Norris[3], Fei Yan[1], David K. Kim[4], Jack Y. Qiu[1,2], Uwe von Lüpke[1], Jonilyn L. Yoder[4], Terry P. Orlando[1,2], Simon Gustavsson[1], Lorenza Viola[3], and William D. Oliver[1,2,4,5]

[1]Research Laboratory of Electronics, Massachusetts Institute of Technology, Cambridge, MA 02139, USA
[2]Department of Electrical Engineering and Computer Science, Massachusetts Institute of Technology, Cambridge, MA 02139, USA
[3]Department of Physics and Astronomy, Dartmouth College, Hanover, NH 03755, USA
[4]MIT Lincoln Laboratory, 244 Wood Street, Lexington, MA 02421, USA
[5]Department of Physics, Massachusetts Institute of Technology, Cambridge, MA 02139, USA

Contents

Supplementary Note 1 Device parameters and fabrication of the qubit sensor 1

Supplementary Note 2 Randomized benchmarking of single-qubit gates 1

Supplementary Note 3 Measurement setup 1

Supplementary Note 4 Control pulse sequences 4

Supplementary Note 5 Monte Carlo simulations 4

Supplementary Note 6 Estimation of the noise mean 5

Supplementary Note 7 PSD estimation procedure 7

Supplementary Note 8 Bispectrum estimation procedure 8

Supplementary Note 1 Device parameters and fabrication of the qubit sensor

Device parameters are summarized in Table 1.

Supplementary Table 1. Device parameters.

Parameter	Value
Qubit frequency $\omega_0/2\pi$	2.920 GHz
Qubit anharmonicity $A/2\pi$	1.163 GHz
Relaxation time T_1	27.0 ± 2.7 µs
Spin-echo relaxation time T_2	35.9 ± 4.4 µs
Free induction decay time T_2^*	12.2 ± 1.0 µs
Readout cavity frequency $\omega_r/2\pi$	7.348 GHz
Readout cavity linewidth $\kappa/2\pi$	2.548 MHz
Dispersive coupling strength $\chi/2\pi$	0.130 MHz

The device was fabricated in the same way as in Ref. [1]. It is a generalized version of the capacitively shunted flux qubit [1], comprising a capacitively shunted small-area junction in parallel is a series array of N junctions. In the capacitively shunted flux qubit of Ref. [1], this array comprised $N = 2$ junctions. Here, the number of array junctions is $N = 8$, far fewer than used in the fluxonium regime of operation [S1]. The area of each Josephson junction forming the array is identical and designed to be $0.2 \times 1.2\ \mu\text{m}^2$. The left junction in Fig. 1a is smaller in area by a factor of 8 than the right junction ($\alpha = 1/8$). The critical current density J_c is measured to be $0.60 \pm 0.01\ \mu\text{A}/\mu\text{m}^2$ and the shunt capacitance C_sh is designed to be 20 fF.

Supplementary Note 2 Randomized benchmarking of single-qubit gates

We characterized an average error rate of single-qubit gates by performing Clifford randomized benchmarking [S2] (Fig. 1). As mentioned in the main text, single-qubit operations are performed using cosine-shaped microwave pulses, applying a quadrature correction (DRAG [2]) to minimize unwanted phase evolution and leakage due to the presence of higher levels.

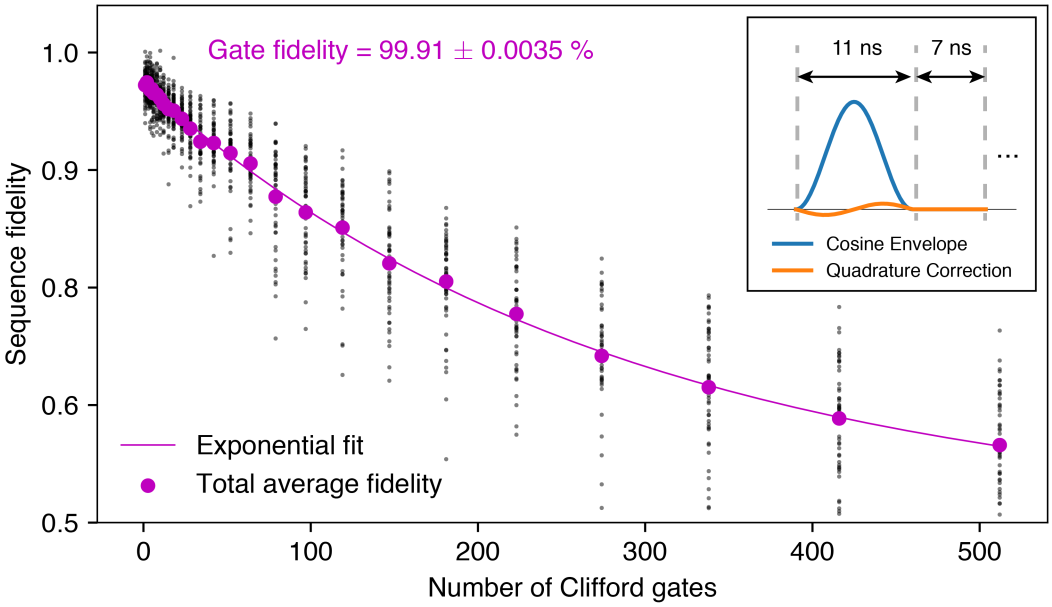

Supplementary Figure 1. Randomized benchmarking of single-qubit gates. Standard single-qubit Clifford randomized benchmarking, indicated as average sequence fidelity (magenta circle) vs. number of Clifford gates. There are 50 randomizations for each number of Clifford gates. The envelope of microwave pulse is a cosine with a total length of 11 ns; a constant buffer time of 7 ns is inserted after each pulse to ensure complete separation of the pulses (inset).

Supplementary Note 3 Measurement setup

3.1 Cryogenic setup

The experiments were performed using a Leiden CF-450 dilution refrigerator, capable of cooling to a base temperature of 15 mK. The samples were magnetically shielded with a superconducting can surrounded by a Cryoperm-10 cylinder. The schematic of the cryogenic circuitry is shown in Figure 2. There are two RF lines for the input and the output of the samples; applying microwave readout tone and measuring the transmission of sample respectively. Thermal noise from room temperature on the

RF drive lines is attenuated with 20 dB at the 3 K stage, followed by 6 dB at still, and 26 dB at the 20 mK stage. All attenuators in the cryogenic samples are made by XMA. Note that there is one additional RF line for pumping the Josephson traveling wave parametric amplifier (JTWPA) [S3] used as a first-stage pre-amplifier to amplify the readout signal at base temperature. The effective noise temperature is determined primarily by the JTWPA, with a total system noise measured to be about 600 mK. To avoid any back-action of the pump-signal from TWPA, we added a microwave isolator between the samples and the TWPA. On the RF output line, there is a high-electron mobility transistor (HEMT) amplifier (Cryo-1-12 SN508D) at the 3 K stage. Two microwave isolators allow for the signal to pass through to the amplifier without being attenuated, while taking all the reflected noise off of the amplifier and dumping it in a 50 Ω termination instead of reaching the sample.

There are two additional lines for qubit flux bias: one is for DC flux bias, which is applied globally through a coil installed in the device package, the other is to apply magnetic flux to the qubit thorough a local antenna. The primary requirement of the DC flux bias line is the ability to tune through at least a single flux quantum on the SQUID of the qubit with high precision and low noise. The local flux bias line is attenuated by 20 dB at the 3 K stage, 6 dB at the still, 20 dB at the 20 mK stage to remove excess thermal photons from higher-temperature stages.

3.2 Room temperature control

Outside of the cryostat, we have all of the control components which allow us to apply microwave signals that address the cavity and the qubits, as well as the components necessary to resolve the readout signal. All the signals are added using microwave power splitters (Marki PD0R413) used in reverse. Direct digital synthesis of the qubit signals is performed using a high-speed arbitrary waveform generator (AWG Keysight M8195A), which has a 65 GS/sec sampling rate and sufficient bandwidth for this purpose. The output line is further amplified outside of the cryostat with an amplifier (MITEQ AMF-5D-00101200-23-10P) with a quoted noise figure of 2.3 dB, and a preamplifier (Stanford Research SR445A). A detailed schematic is given in Fig 2. We use an IQ demodulation technique to mix down the signal entering the RF port with a reference signal detuned by 40 MHz applied to the LO port. This results in down-converted signals to 40 MHz using a mixer. All components are frequency-locked via a common SRS rubidium frequency standard (10 MHz).

3.3 Pulse generation

Qubit control pulse generation is performed via a Keysight M8195A AWG. The pulses are programmed in Labber and then uploaded to the Keysight M8195A.

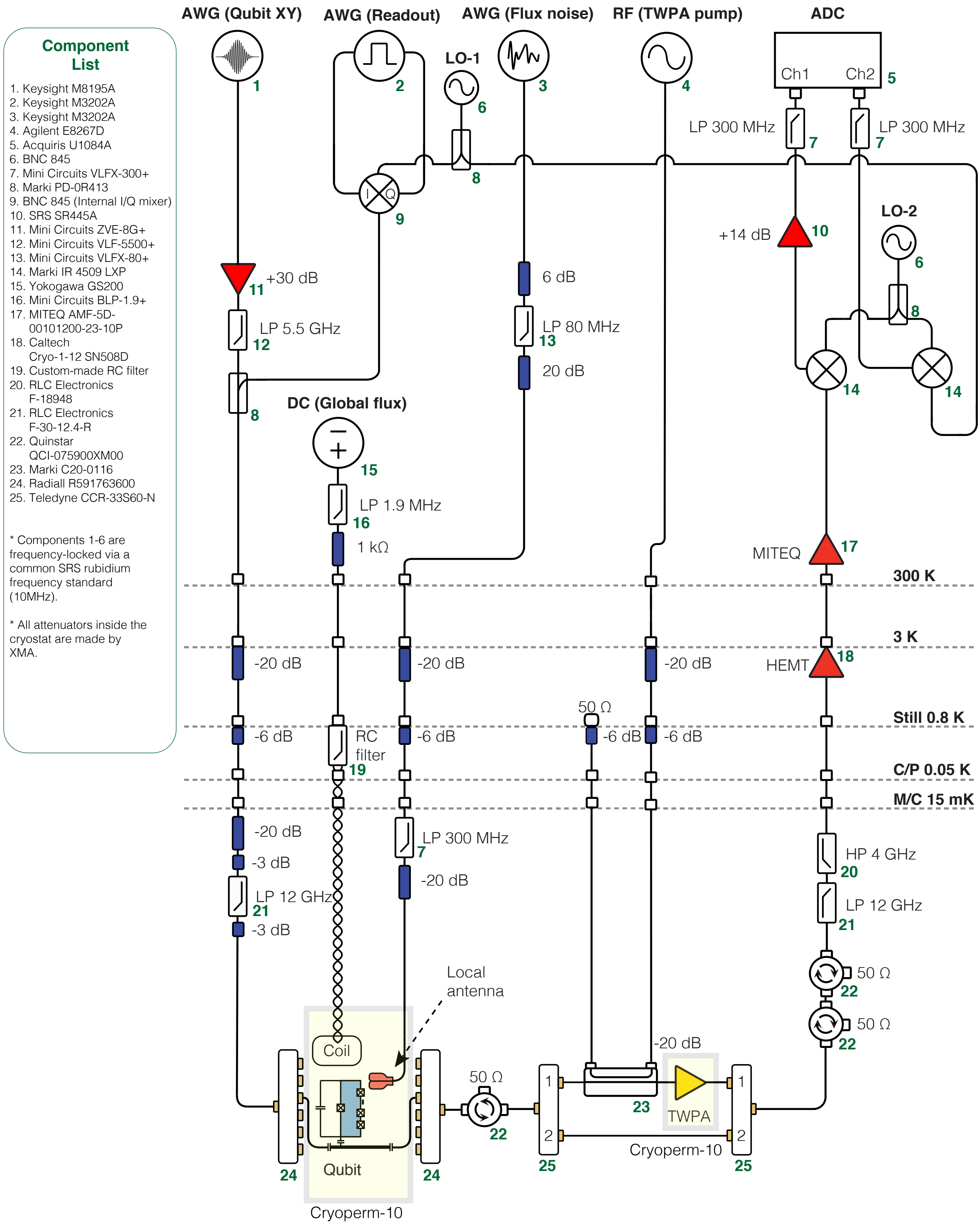

Supplementary Figure 2. Electronics and control wiring

3.4 Generation of engineered noise

To synthesize a zero-mean flux-noise process $\Delta\Phi(t) \equiv \Phi(t) - \Phi_0/2$ with a given PSD, we use an AWG to produce sample waveforms consisting of N_h harmonics

$$\Delta\Phi(t) = \sum_{m=1}^{N_h} (a_m \cos\omega_m t + b_m \sin\omega_m t)\,, \tag{1}$$

where $\omega_m = 2\pi m/T_0$, and with $2\pi/T_0$ the fundamental angular frequency. The Fourier coefficients a_m and b_m are random variables with

$$\mathbb{E}[a_m] = \mathbb{E}[b_m] = 0, \quad \forall\, m, \qquad \mathbb{E}[a_m b_n] = 0, \quad \forall m, n, \qquad \mathbb{E}[a_m a_n] = \mathbb{E}[b_m b_n] = 0, \quad \forall\, m \neq n. \tag{2}$$

Further taking a_m and b_m to have normal (Gaussian) distributions with variance $\sigma_m^2 = 2S_\Phi(\omega_m)/T_0$, the waveforms $\Delta\Phi(t)$ become a discrete approximation of a Gaussian stochastic process with a frequency-domain PSD $S_\Phi(\omega)$.

In all experiments presented in the main text, we consider

$$S_\Phi(\omega) = \frac{P_0/\pi\omega_c}{1 + (\omega/\omega_c)^2}. \tag{3}$$

with P_0 the noise power and $\omega_c/2\pi = 0.5$ MHz. For the experiment presented in Fig. 2 of the main text, to produce a discrete approximation of a noise process with this spectrum, we take $T_0 = 20$ μs and $N_h = 10^3$, corresponding to harmonics separated by the fundamental frequency $1/T_0 = 50$ kHz with a high-frequency cutoff at $\omega_{N_h}/2\pi = N_h/T_0 = 50$ MHz. For the experiment presented in Figs. 4 and 5 of the main text, we take $T_0 = 200$ μs and $N_h = 10^4$, yielding harmonics separated by $1/T_0 = 5$ kHz with the same high-frequency cutoff at $\omega_{N_h}/2\pi = N_h/T_0 = 50$ MHz. A new waveform is produced by the AWG for each measurement of a Pauli operator performed on the qubit to ensure statistical independence of the samples of the stochastic process, leading to a total number of noise samples of 40,000 for the experiment presented in Fig. 2, and 80,000 for Figs 4 and 5. The waveforms have a duration of 1.25 μs for the experiment presented in Fig. 2 and 20 μs for Figs. 4 and 5.

Supplementary Note 4 Control pulse sequences

The set of control pulse sequences designed for reconstructing the bispectrum are summarized in Table 2 and visualized in Fig. 3. Note that all control pulse sequences start and end with a $\pi/2$ pulse for the purposes of state preparation and tomography.

Supplementary Table 2. Control pulse sequences designed for non-Gaussian spectral estimation.

Seq. Index p	Position of π pulses [ns]	Repetitions M	Filter function at zero frequency $F_p(0,T)$
1	No pulses (free evolution)	1	$\neq 0$
2	125, 175, 225, 275, 325, 610, 820, 875	10	$\neq 0$
3	90, 235, 410, 555, 730, 875	10	$\neq 0$
4	80, 150, 205, 355, 560, 630, 685, 835	10	$\neq 0$
5	105, 240, 345, 480, 585, 720, 825, 960	10	$\neq 0$
6	85, 135, 185, 240, 455, 775, 825, 880	10	0
7	130, 180, 285, 335, 475, 765, 870, 960	10	0
8	90, 150, 200, 305, 500, 715, 860, 960	10	0
9	80, 320, 370, 425, 600, 650, 720, 855	10	0
10	205, 310, 360, 545, 645, 725, 850, 960	10	0
11	145, 365, 425, 495, 600, 680, 850, 960	10	0

Supplementary Note 5 Monte Carlo simulations

For the Monte Carlo simulations that are presented in the main text, we consider a single qubit controlled via a microwave drive at angular frequency ω_d, which is used to apply pulses about σ_x and σ_y. In contrast with the main text, here we do not assume that these pulses are instantaneous. To describe the time-evolution of the qubit under the combined action of these finite-width pulses and classical noise described by the process $B(t)$, we consider the Hamiltonian in the lab frame,

$$H(t) = \frac{\omega_\mathrm{q} + B(t)}{2}\sigma_z + \varepsilon(t)\cos[\omega_\mathrm{d} t + \theta(t)]\sigma_x, \tag{4}$$

where ω_q is the qubit angular frequency, and $\varepsilon(t)$ and $\theta(t)$ are the drive amplitude and phase, respectively.

We next move to the frame that rotates at the drive frequency by applying the unitary transformation $R_\mathrm{d}(t) = \exp(-i\omega_\mathrm{d} t\,\sigma_z/2)$, leading to the Hamiltonian $H_\mathrm{d}(t) = R_\mathrm{d}^\dagger(t) H(t) R_\mathrm{d}(t) - iR_\mathrm{d}^\dagger(t)\dot{R}_\mathrm{d}(t)$ in the rotating frame. This gives

$$H_\mathrm{d}(t) = \frac{D + B(t)}{2}\sigma_z + H_\mathrm{c}(t), \tag{5}$$

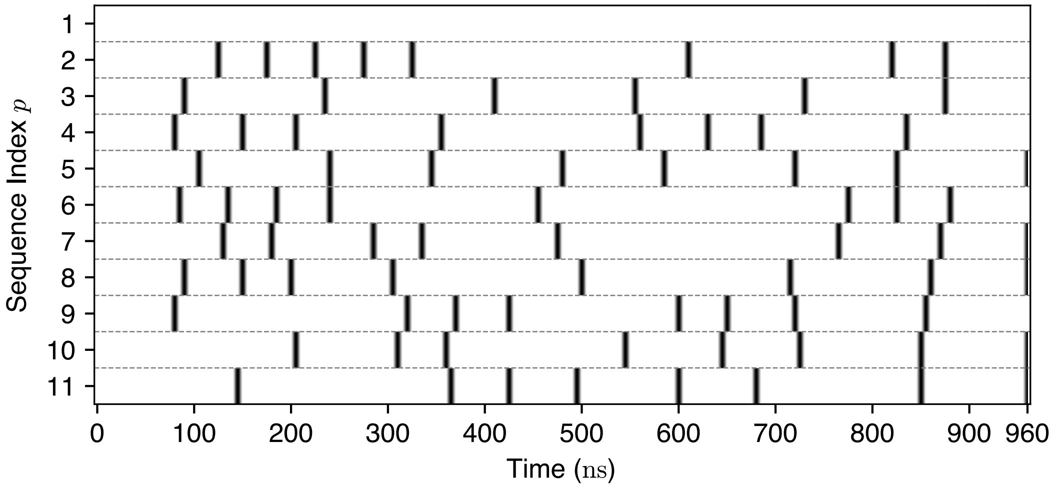

Supplementary Figure 3. Timing diagrams of the base control sequences. Only π pulses are shown.

where $D \equiv \omega_{\mathrm{q}} - \omega_{\mathrm{d}}$ is the drive detuning and $H_{\mathrm{c}}(t) \equiv \varepsilon(t)\cos[\omega_{\mathrm{d}}t + \theta(t)][\sigma_+ \exp(i\omega_{\mathrm{d}}t) + \mathrm{H.c.}]$ is the control Hamiltonian. Assuming that $\varepsilon(t) \ll \omega_{\mathrm{d}}$ and that $\theta(t)$ varies on a timescale much longer than $2\pi/\omega_{\mathrm{d}}$ allows us to invoke the rotating-wave approximation, under which terms oscillating like $\exp\{\pm[2i\omega_{\mathrm{d}}t + \theta(t)]\}$ are neglected. The resulting control Hamiltonian may be simplified as

$$H_{\mathrm{c}}(t) \approx \frac{1}{2}\left[\eta_{\mathrm{I}}(t)\sigma_x + \eta_{\mathrm{Q}}(t)\sigma_y\right], \tag{6}$$

where $\eta_{\mathrm{I}}(t) \equiv \varepsilon(t)\cos\theta(t)$ and $\eta_{\mathrm{Q}}(t) \equiv \varepsilon(t)\sin\theta(t)$ are the envelopes of the in-phase and quadrature components of the microwave control signal, respectively. Equation (6) then describes finite-width pulses about x or y axes.

We perform Monte Carlo simulations by solving the time-dependent Schrödinger equation associated with the Hamiltonian $H_{\mathrm{d}}(t)$ given by Eq. (5), under the control Hamiltonian of Eq. (6). The drive detuning D is set to zero in the simulations. The envelope of each control pulse is cosine with total pulse duration 11 ns (see inset of Fig. 1).

In our Monte Carlo simulations, we account for non-Gaussian noise by setting $B(t) \equiv \beta_\Phi \Delta\Phi(t)^2$ in Eq. (5), and producing random samples of Gaussian flux-noise $\Delta\Phi(t)$ through the approach described in Section 3.4 of this Supplement. Because this approach relies on an exact solution of the qubit evolution under the noise samples, it is equivalent to accounting for all the terms in the cumulant expansion. The fundamental frequency $1/T_0$ and the number of harmonics N_h involved in the Fourier-series representation of the noise process are the same as in Section 3.4. To perform the simulations, we generate 100,000 noise samples for the data presented in Fig. 2 of the main text, and 80,000 noise samples for Figs. 4 and 5.

Supplementary Note 6 Estimation of the noise mean

6.1 Ramsey estimation protocol

To measure the noise mean μ_B, we use the Ramsey sequence illustrated in Fig. 4a. In this sequence, $\pi/2$ pulses about σ_x and σ_y are applied at times $t = 0$ and $t = T$, respectively, followed by a measurement of the qubit in the σ_z eigenbasis at time $t_f = T + \Delta T$, where ΔT is a buffer time much shorter than T, but longer than the pulse width. To lay down the theoretical basis of the procedure, we start from the rotating-frame Hamiltonian $H_{\mathrm{d}}(t)$ introduced in Eq. (5), above. To describe the effects of control with finite-width pulses, it is useful to move to the toggling frame using the unitary transformation $R_{\mathrm{T}}(t) = \mathcal{T}\exp[-i\int_0^t ds\, H_{\mathrm{c}}(s)]$, with $\mathcal{T}$ the time-ordering operator, and where $H_c(t)$ is given by Eq. (6). In this toggling frame, the Hamiltonian is

$$H_{\mathrm{T}}(t) = \frac{D + B(t)}{2}\vec{y}_p(t)\cdot\vec{\sigma}, \qquad \vec{\sigma} \equiv (\sigma_x, \sigma_y, \sigma_z), \tag{7}$$

where $\vec{y}_p(t)$ has components $y_{p,a}(t) \equiv \frac{1}{2}\mathrm{Tr}[R_{\mathrm{T}}^\dagger(t)\sigma_z R_{\mathrm{T}}(t)\sigma_a]$, with $a \in \{x, y, z\}$, for pulse sequence p. Remark that a pulse sequence consisting of instantaneous π pulses (instead of the Ramsey sequence considered here) would result in $\vec{y}_p(t) = [0, 0, y_p(t)]$, where $y_p(t)$ is the switching function used in the main text.

Moving back to the lab frame, the expectation of the z component of the qubit polarization after the pulse sequence is

$$\langle\sigma_z(t_f)\rangle = \mathbb{E}\left\{\mathrm{Tr}\left[R_{\mathrm{T}}^\dagger(t_f)\sigma_z R_{\mathrm{T}}(t_f)U_{\mathrm{T}}(t_f)\rho_0 U_{\mathrm{T}}^\dagger(t_f)\right]\right\}, \tag{8}$$

where $U_{\mathrm{T}}(t) \equiv \mathcal{T}\exp[-i\int_0^t ds\, H_{\mathrm{T}}(s)]$ is the time-evolution operator in the toggling frame, and ρ_0 is the initial qubit density matrix, before application of the pulses. We evaluate $\langle\sigma_z(t_f)\rangle$ perturbatively by performing a Dyson expansion of $U_{\mathrm{T}}(t)$.

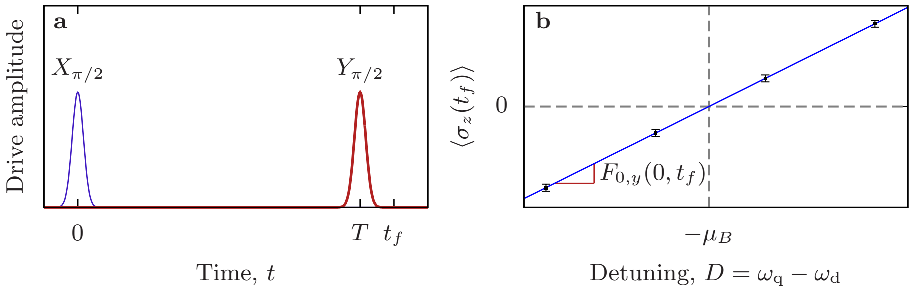

Supplementary Figure 4. Ramsey protocol for estimation of the noise mean. a, Envelopes $\eta_I(t)$ (thin blue line) and $\eta_Q(t)$ (thick red line) of the in-phase and quadrature components of the $\pi/2$ pulses applied about σ_x and σ_y, respectively (see Eq. (6) in the text). **b**, Illustration of the technique for estimation of the mean by linear regression. Black error bars: Monte Carlo simulations of the z component of the qubit polarization $\langle\sigma_z(t_f)\rangle$ after the pulse sequence as a function of the detuning of the drive from the qubit frequency. Blue line: linear regression. According to Eq. (10), the x-intercept of the blue line gives $-\mu_B$, and its slope gives the filter function $F_{0,y}(0,t_f)$, defined below Eq. (9).

Assuming that t_f is sufficiently short and that $D + B(t)$ is sufficiently small, we truncate this expansion to the first order in $D + B(t)$. Upon substitution into Eq. (8), this approach is equivalent to neglecting any contribution of cumulants of the noise beyond order 1. Taking $\rho_0 \equiv |0\rangle\langle 0|$, with $|0\rangle$ the eigenstate of σ_z with eigenvalue -1, then yields

$$\langle\sigma_z(t_f)\rangle \approx -y_{p,z}(t_f) + (D+\mu_B)\left[F_{p,x}(0,t_f)y_{p,y}(t_f) - F_{p,y}(0,t_f)y_{p,x}(t_f)\right], \tag{9}$$

where we have introduced the filter functions $F_{p,a}(\omega,t) \equiv \int_0^t ds\, e^{-i\omega s} y_{p,a}(s)$, with $a \in \{x,y,z\}$. For the pulse sequence illustrated in Fig. 4a (which we label by $p = 0$), neglecting any overlap between the pulses, it is straightforward to show that $\vec{y}_p(t_f) \equiv \vec{y}_0(t_f) = (-1,0,0)$ for $t_f = T + \Delta t$, leading to

$$\langle\sigma_z(t_f)\rangle \approx (D+\mu_B)T', \tag{10}$$

where $T' \equiv F_{0,y}(0,t_f) = \int_0^{t_f} ds\, y_{0,y}(s)$ can be viewed as an effective pulse interval accounting for the shape of the pulses. For instantaneous pulses, $T' = T$.

According to Eq. (10), setting $D = 0$, the first noise cumulant $C^{(1)}(0) \equiv \mu_B$ may be estimated simply by measuring $\langle\sigma_z(t_f)\rangle$ and evaluating $\langle\sigma_z(t_f)\rangle/F_{0,y}(0,t_f)$. However, this approach requires accurate knowledge of $F_{0,y}(0,t_f)$, and thus of the shape of the control pulses. For the short Ramsey sequences required here to neglect cumulants of order higher than one in Eqs. (9)-(10), the estimate of μ_B then becomes excessively sensitive to distortions of the pulse envelopes that occur in practice. As a result, estimates of μ_B become significantly biased. Since the bispectrum estimation technique that will be discussed in Sec. Supplementary Note 8 requires precise knowledge of μ_B, this bias precludes accurate non-Gaussian QNS.

6.2 Robust implementation via linear regression

Crucially, the vulnerability of the above Ramsey scheme to pulse-width effects can be alleviated by estimating μ_B with a linear regression procedure. Indeed, according to Eq. (10), plotting $\langle\sigma_z(t_f)\rangle$ as a function of the detuning D results in a straight line that intersects with the abscissa at $D = -\mu_B$ (Fig. 4b), *irrespective of* $F_{0,y}(0,t_f)$. Therefore, measuring $\langle\sigma_z(t_f)\rangle$ as a function of D and performing a linear fit of the resulting data leads to an estimate of μ_B that is insensitive to the pulse shape to the first order in $D + B(t)$.

To apply this idea to our experimental data, we now explicitly construct an estimator of μ_B based on linear regression. For each drive detuning D_j, with $j \in \{1,2,\ldots,N_D\}$, we consider N projective measurements of σ_z yielding outcomes $Z_{j,i} = +1$ or $Z_{j,i} = -1$, where i labels measurements. In the limit $N \gg 1$, the sample mean $\overline{Z}_j$ of the projective measurements for detuning D_j becomes Gaussian distributed,

$$\overline{Z}_j \equiv \frac{1}{N}\sum_{i=1}^{N} Z_{j,i} \sim \text{Normal}\left[(D_j+\mu_B)F_{0,y}(0,t_f),\ \frac{\text{var}(\sigma_z)_j}{N}\right], \tag{11}$$

where $\text{var}(\sigma_z)_j \equiv \langle\sigma_z(t_f)^2\rangle_j - \langle\sigma_z(t_f)\rangle_j^2$ is the expected variance of σ_z averaged over realizations of the noise process, for detuning D_j. Assuming that $\text{var}(\overline{Z}_j) = \text{var}(\sigma_z)_j/N$ is the same for all relevant detunings, $\text{var}(\overline{Z})_j \equiv \text{var}(\overline{Z})\,\forall\, j$, Eq. (11) then corresponds to the *conditional normal model of linear regression* [S4], in which deviations of the data points from the expected linear behavior are given by independent and identically distributed (i.i.d.) Gaussian random variables. This assumption of uniform variance is justified in an approximate sense for ideal projective measurements. Indeed, in this situation, $\text{var}(\overline{Z}_j) = [1-\langle\sigma_z(t_f)\rangle_j^2]/N$, so that $\text{var}(\overline{Z}_j)$ is independent of D_j to first order in $\langle\sigma_z(t_f)\rangle_j \approx (D+\mu_B)F_{0,y}(0,t_f)$, with $\text{var}(\overline{Z}_j) \approx 1/N$. Limiting ourselves to detunings for which $\langle\sigma_z(t_f)\rangle_j \lesssim 0.05$ (see Fig. 4a), we find that $\text{var}(\overline{Z}_j)$ (estimated from the sample mean of measurements of σ_z) varies by less than 5% across values of D_j.

To define our estimator of μ_B within the conditional normal model of linear regression, we first introduce the quantities $a \equiv \mu_B F_{0,y}(0,t_f)$ and $b \equiv F_{0,y}(0,t_f)$, corresponding to the y-intercept and slope of the linear equation $\langle\sigma_z(t_f)\rangle_j = (D_j + \mu_B)F_{0,y}(0,t_f) \equiv a + b\,D_j$, respectively. Maximizing the likelihood of a and b with respect to measurement outcomes $\overline{Z}_j$ with

the probability distribution given by Eq. (11) then yields the estimators

$$a^{\rm est} = N_D^{-1} \sum_{j=1}^{N_D} \left(\overline{Z}_j - b^{\rm est} D_j\right), \tag{12}$$

$$b^{\rm est} = \frac{\sum_j \left(D_j - N_D^{-1}\sum_k D_k\right)\left(\overline{Z}_j - N_D^{-1}\sum_k \overline{Z}_k\right)}{\sum_j \left(D_j - N_D^{-1}\sum_k D_k\right)^2}. \tag{13}$$

The estimators defined by Eqs. (12) and (13) are Gaussian random variables with $E(a^{\rm est}) = a$, $E(b^{\rm est}) = b$, and

$$\mathrm{var}(a^{\rm est}) = \frac{N_D^{-1}\sum_j D_j^2}{\sum_j \left(D_j - N_D^{-1}\sum_k D_k\right)^2}\mathrm{var}(\overline{Z}), \qquad \mathrm{var}(b^{\rm est}) = \frac{\mathrm{var}(\overline{Z})}{\sum_j \left(D_j - N_D^{-1}\sum_k D_k\right)^2},$$

$$\mathrm{cov}(a^{\rm est}, b^{\rm est}) \equiv \mathbb{E}[(a^{\rm est} - a)(b^{\rm est} - b)] = -\frac{N_D^{-1}\sum_j D_j}{\sum_j \left(D_j - N_D^{-1}\sum_k D_k\right)^2}\mathrm{var}(\overline{Z}).$$

To estimate μ_B, we use

$$\tilde{\mu}_B^{\rm est} \equiv a^{\rm est}/b^{\rm est}. \tag{14}$$

When $\mathrm{var}(a^{\rm est})^{1/2}$ and $\mathrm{var}(b^{\rm est})^{1/2}$ are sufficiently small, we expand $\tilde{\mu}_B^{\rm est}$ in powers of $\delta a^{\rm est} \equiv a^{\rm est} - a$ and $\delta b^{\rm est} \equiv b^{\rm est} - b$. Truncating to the first order in $\delta a^{\rm est}$ and $\delta b^{\rm est}$, $\tilde{\mu}_B^{\rm est}$ becomes approximately Gaussian-distributed with $\mathbb{E}(\tilde{\mu}_B^{\rm est}) \approx \mu_B$ and

$$\mathrm{var}(\tilde{\mu}_B^{\rm est}) \approx \frac{b^2\,\mathrm{var}(a^{\rm est}) + a^2\,\mathrm{var}(b^{\rm est}) - 2ab\,\mathrm{cov}(a^{\rm est}, b^{\rm est})}{b^4}. \tag{15}$$

For the experimental data presented in Fig. 4a of the main text, $\mathrm{var}(\tilde{\mu}_B^{\rm est})$ is estimated by replacing $a \to a^{\rm est}$ and $b \to b^{\rm est}$ in Eq. (15), with $a^{\rm est}$ and $b^{\rm est}$ given by Eqs. (12) and (13), respectively.

Finally, to isolate the shift in the qubit frequency due to the first cumulant of the engineered source of noise, we apply the above procedure first in the absence of noise, and then in its presence. This yields the two sets of data points shown in Fig. 4a of the main text. Fitting a straight line through each data set and substracting their x-intercept then gives our final estimate of μ_B, $\mu_B^{\rm est} = \mu_B^{\rm on} - \mu_B^{\rm off}$, where $\mu_B^{\rm on(off)}$ is the estimator defined by Eq. (14) with ($\mu_B^{\rm on}$) or without ($\mu_B^{\rm off}$) engineered noise. The variance of $\mu_B^{\rm est}$ is then simply $\mathrm{var}(\mu_B^{\rm est}) = \mathrm{var}(\mu_B^{\rm on}) + \mathrm{var}(\mu_B^{\rm off})$. For the experimental data presented in the main text, we find $\mu_B^{\rm est}/2\pi = 127.1$ kHz with a standard deviation $\mathrm{var}(\mu_B^{\rm est})^{1/2} = 3.86$ kHz, corresponding to the 95% confidence interval $\mu_B^{\rm est}/2\pi = 127.1 \pm 7.56$ kHz.

Supplementary Note 7 PSD estimation procedure

To estimate the PSD, we build on the frequency-comb approach introduced by Alvarez and Suter in Ref. [3]. As detailed in the main text, treating the FFs as frequency combs generates a system of linear equations, solving which determines the PSD sampled at the harmonic frequencies. Rather than solving this sytem by matrix inversion as in Ref. [3], we employ a statistically-motivated maximum likelihood estimate (MLE), which takes experimental error into account. The likelihood function we use follows from the asymptotic Gaussian distribution of the measured decay constants, which we describe in Sec. 7.1. In Sec. 7.2, we determine the likelihood, the conditional probability of obtaining a set of decay data conditioned on the actual value of the PSD. The task of maximizing the likelihood can be cast as a linear problem, allowing clear comparison with Ref. [3].

7.1 Distribution of the decay constant

The PSD enters the dynamics of the qubit through the decay constant in Eq. (1), which can be obtained from measurements of the transverse Pauli operators, σ_x and σ_y. Let $\sigma_i^{\rm est}$ denote the estimated expected value of σ_i for $i \in \{x, y\}$ after the qubit has evolved for a time t under control sequence p. In the limit of a large number of measurements, $\sigma_i^{\rm est}$ is approximately Gaussian distributed with mean $\mu_i = \mathbb{E}[\langle\sigma_i(t)\rangle]$ and variance $\mathrm{var}[\sigma_i^{\rm est}]$. In terms of the estimated expected values, the estimated decay constant is

$$\chi_p^{\rm est}(t) = -\frac{1}{2}\ln(\sigma_x^{\rm est\,2} + \sigma_y^{\rm est\,2}) = -\frac{1}{2}\ln\left[(\tilde{\sigma}_x^{\rm est} + \mu_x)^2 + (\tilde{\sigma}_y^{\rm est} + \mu_y)^2\right], \tag{16}$$

where $\tilde{\sigma}_i^{\rm est} = \sigma_i^{\rm est} - \mu_i$. Note that $\sigma_i^{\rm est}$, μ_i and $\tilde{\sigma}_i^{\rm est}$ on the right side of this expression depend implicitly on the time t. When $\mathrm{var}[\sigma_x^{\rm est}]$, $\mathrm{var}[\sigma_y^{\rm est}] \ll 1$, we can expand this expression about $\tilde{\sigma}_x^{\rm est}, \tilde{\sigma}_y^{\rm est} \approx 0$, yielding

$$\chi_p^{\rm est}(t) \approx -\frac{1}{2}\ln(\mu_x^2 + \mu_y^2) - \left(\frac{\mu_y}{\mu_x^2 + \mu_y^2}\right)\tilde{\sigma}_y^{\rm est} - \left(\frac{\mu_x}{\mu_x^2 + \mu_y^2}\right)\tilde{\sigma}_x^{\rm est}.$$

Since it is a linear combination of Gaussian distributed random variables, the decay constant is also Gaussian distributed with mean and variance,

$$\mathbb{E}[\chi_p^{\text{est}}(t)] = -\frac{1}{2}\ln(\mu_x^2 + \mu_y^2) = \frac{1}{2\pi}\int_{-\infty}^{\infty} d\omega |F_p(\omega,t)|^2 S(\omega) + \Theta(t^4) \tag{17}$$

$$\text{var}[\chi_p^{\text{est}}(t)] = \left(\frac{\mu_y}{\mu_x^2+\mu_y^2}\right)^2 \text{var}[\sigma_y^{\text{est}}] + \left(\frac{\mu_x}{\mu_x^2+\mu_y^2}\right)^2 \text{var}[\sigma_x^{\text{est}}]. \tag{18}$$

7.2 Maximum likelihood estimate

Recall from the main text that after $M \gg 1$ repetitions of a control sequence p with cycle time T, the FF in Eq. (17) is an approximate frequency comb, enabling us to write the decay constant as

$$\mathbb{E}[\chi_p^{\text{est}}(MT)] \approx \frac{M}{T}\sum_{k=-\infty}^{\infty} |F_p(k\omega_h, T)|^2 S(k\omega_h).$$

Using the symmetry $S(\omega) = S(-\omega)$, and the decay of the PSD and FF at high frequencies, we can truncate the sum above to a finite number of harmonic frequencies,

$$\mathbb{E}[\chi_p^{\text{est}}(MT)] \approx \frac{M}{T}\sum_{k=0}^{K-1} \Big(\frac{2-\delta_{k,0}}{2}\Big)|F_p(k\omega_h, T)|^2 S(k\omega_h).$$

From the expected value above and the variance in Eq. (18), the conditional probability of measuring $\chi_p^{\text{est}}(MT)$ given the actual values of the PSD, $\vec{S} = [S(0), \ldots, S(K\omega_h)]^T$, is

$$P\big[\chi_p^{\text{est}}(MT)|\vec{S}\big] = \frac{1}{\sqrt{2\pi\,\text{var}[\chi_p^{\text{est}}(MT)]}} \exp\left\{ -\frac{\Big[\chi_p^{\text{est}}(MT) - \frac{M}{T}\sum_{k=0}^{K-1}\Big(\frac{2-\delta_{k,0}}{2}\Big)|F_p(k\omega_h,T)|^2 S(k\omega_h)\Big]^2}{2\,\text{var}[\chi_p^{\text{est}}(MT)]} \right\}. \tag{19}$$

The estimate of the PSD is based on the likelihood or conditional probability of measuring $\vec{\chi} = [\chi_1^{\text{est}}(MT), \ldots, \chi_P^{\text{est}}(MT)]^T$ for a set of control sequences $p = 1, \ldots, P$ with $P \geq K$. Since the measurements of the decay constant are uncorrelated, the likelihood follows from Eq. (19),

$$P(\vec{\chi}|\vec{S}) = \prod_{p=1}^{P} P\big[\chi_p^{\text{est}}(MT)|\vec{S}\big] = \big[(2\pi)^P \det \mathbf{\Sigma}\big]^{-\frac{1}{2}} \exp\left[-\frac{1}{2}(\vec{\chi} - \mathbf{B}\vec{S})^T \mathbf{\Sigma}^{-1}(\vec{\chi} - \mathbf{B}\vec{S})\right], \tag{20}$$

where the $P \times P$ covariance matrix has elements $\mathbf{\Sigma}_{p,q} = \text{var}[\chi_p^{\text{est}}(MT)]\,\delta_{p,q}$ and the $P \times K$ reconstruction matrix $\mathbf{B}$ depends on the FFs evaluated at the harmonic frequencies

$$\mathbf{B}_{p,k} = \frac{M}{T}\Big(\frac{2-\delta_{k,0}}{2}\Big)|F_p(k\omega_h, T)|^2.$$

Since the likelihood is Gaussian, the maximum likelihood estimate of the PSD is equivalent to the value of $\vec{S}$ that minimizes the argument of the exponential in Eq. (20),

$$\vec{S}^{\text{MLE}} = \underset{\vec{S}}{\text{argmin}}\, \frac{1}{2}(\vec{\chi} - \mathbf{B}\vec{S})^T \mathbf{\Sigma}^{-1}(\vec{\chi} - \mathbf{B}\vec{S}). \tag{21}$$

The least squares estimate of the PSD originally used in Ref. [3], given by $\vec{S}^{\text{LS}} = \mathbf{B}^{-1}\vec{\chi}$, is recovered when $\mathbf{\Sigma} = \mathbf{I}$. This implies that all measurements of the decay constant contribute equally to the estimate. In contrast, the dependence of $\vec{S}^{\text{MLE}}$ on the actual variances in $\mathbf{\Sigma}$ ensures that measurements with more uncertainty contribute less to the estimate.

In the experiment, the PSD is reconstructed at the harmonics $k = 0, \ldots, 7$ using the $P = 11$ control sequences depicted in Fig. (3), all with cycle time $T = 960\,$ns. This differs from the implementation of Ref. [3], which uses CPMG control sequences of varying cycle times. For each control sequence, the transverse Pauli components are measured to obtain σ_x^{est}, σ_y^{est} and $\chi_p^{\text{est}}(MT)$ from Eq. (16). The variances of $\chi_p^{\text{est}}(MT)$, which comprise $\mathbf{\Sigma}$, follow from Eq. (18) with μ_x and μ_y replaced by the estimated values σ_x^{est} and σ_y^{est}. In principle, the use of $P = 11$ control sequences would enable us to reconstruct the PSD at $K = 11$ harmonics. For the particular set of control sequences we used, however, the reconstruction matrix $\mathbf{B}$ becomes ill-conditioned for $K > 8$, limiting the number of reconstructable harmonics.

Supplementary Note 8 Bispectrum estimation procedure

While this work is based on the non-Gaussian QNS protocol originally proposed in Ref. [4], the estimation procedure we implemented contains several innovations aimed at generalizing the noise model and improving robustness in the presence of experimental error and numerical instability. First, the zero-mean, non-Gaussian noise model of Ref. [4] is insufficient to describe

the square noise engineered in our quarton-qubit sensor, which is inherently nonzero mean. This complicates the estimation procedure, since both the bispectrum and the mean enter the qubit dynamics through the phase in Eq. (2). Estimating the bispectrum requires that we disambiguate the phase contribution of the bispectrum from that of the mean, which we accomplish by first estimating the noise mean and then isolating the dynamical contribution of the bispectrum in the non-Gaussian phase. A second key difference is the "single-shot" nature of the current estimation procedure. In Ref. [4], control sequences with non-zero filter order were first used to estimate the bispectrum at "non-zero harmonics", i.e., $(k_1\omega_h, k_2\omega_h)$ for which $k_1, k_2 \neq 0$. This estimate was combined with subsequent phase measurements to estimate the bispectrum at "zero harmonics", i.e. $(k_1\omega_h, k_2\omega_h)$ for which $k_1 = 0$ and/or $k_2 = 0$. In the RMLE estimate of the spectrum presented here, both the zeros and the non-zero harmonics are estimated simultaneously, eliminating any compounding of error that can occur in the two-step procedure. The present work also departs from Ref. [4] significantly in its use of a statistically motivated maximum likelihood estimation procedure. As discussed in the main text, the least-squares estimate of Ref. [4] is susceptible to numerical instability and, additionally, does not take measurement error into account.

In the remainder of this section, we fully detail our bispectrum estimation procedure. We begin by describing the probability distribution of the estimated "non-Gaussian" phase angle (φ_p), which enables us to derive the likelihood function for the probability of obtaining a particular set of phase data conditioned on the actual value of the bispectrum (Subsection 8.3). From the likelihood, the task of reconstructing the bispectrum can be mapped into an RMLE problem, as shown Subsection 8.4. The RMLE approach increases numerical stability, accounts for experimental error and allows us to deploy prior knowledge of the bispectrum in the estimation procedure. Since regularization can introduce error into the estimate if it is too strong, in Subsection 8.5 we determine an appropriate regularization strength for our problem using the L-curve criterion.

8.1 Distribution of the non-Gaussian phase

The non-Gaussian phase of the qubit is determined from the estimated expected values of the transverse Pauli operators, σ_x^{est} and σ_y^{est}, when the qubit has evolved under control sequence p for a time t. Recall from Sec. Supplementary Note 7 that in the limit of a large number of measurements, σ_i^{est} is approximately Gaussian distributed with mean $\mu_i = \mathbb{E}[\langle\sigma_i(t)\rangle]$ and variance var$[\sigma_i^{\text{est}}]$. From the estimated expected values, the ordinary phase is determined by

$$\phi_p^{\text{est}}(t) = -\tan^{-1}\left(\frac{\sigma_x^{\text{est}}}{\sigma_y^{\text{est}}}\right) = -\tan^{-1}\left(\frac{\tilde{\sigma}_x^{\text{est}} + \mu_x}{\tilde{\sigma}_y^{\text{est}} + \mu_y}\right), \quad (22)$$

where σ_i^{est}, μ_i and $\tilde{\sigma}_i^{\text{est}} = \sigma_i^{\text{est}} - \mu_i$ depend implicitly on the time t. When var$[\sigma_x^{\text{est}}]$, var$[\sigma_y^{\text{est}}] \ll 1$, we can expand ϕ_p^{est} about $\tilde{\sigma}_x^{\text{est}}$, $\tilde{\sigma}_y^{\text{est}} \approx 0$, yielding

$$\phi_p^{\text{est}}(t) \approx -\tan^{-1}\left(\frac{\mu_x}{\mu_y}\right) - \left(\frac{\mu_y}{\mu_x^2 + \mu_y^2}\right)\tilde{\sigma}_x^{\text{est}} + \left(\frac{\mu_x}{\mu_x^2 + \mu_y^2}\right)\tilde{\sigma}_y^{\text{est}}.$$

As a linear combination of Gaussian distributed random variables, the phase is also Gaussian distributed with mean and variance

$$\mathbb{E}[\phi_p^{\text{est}}(t)] = -\tan^{-1}\left(\frac{\mu_x}{\mu_y}\right) = F_p(0,t)\mu_B - \frac{1}{3!(2\pi)^2}\int_{\mathbb{R}^2} d\vec{\omega}\, G_p(\vec{\omega}, t)\, S_2(\vec{\omega}) + \Theta[t^5], \quad (23)$$

$$\text{var}[\phi_p^{\text{est}}(t)] = \left(\frac{\mu_y}{\mu_x^2 + \mu_y^2}\right)^2 \text{var}[\sigma_x^{\text{est}}] + \left(\frac{\mu_x}{\mu_x^2 + \mu_y^2}\right)^2 \text{var}[\sigma_y^{\text{est}}].$$

The second equality on the right-hand side of $\mathbb{E}[\phi_p^{\text{est}}]$ follows from Eq. (2). Subtracting out the contribution of the noise mean from the phase produces the non-Gaussian phase,

$$\varphi_p^{\text{est}}(t) = \phi_p^{\text{est}}(t) - F_p(0,t)\mu_B^{\text{est}}, \quad (24)$$

where μ_B^{est} is the estimated noise mean described in Sec. Supplementary Note 6. Using the asymptotic Gaussian distribution of μ_B^{est} with mean μ_B and variance var$[\mu_B^{\text{est}}]$, the non-Gaussian phase is similarly Gaussian with mean and variance

$$\mathbb{E}[\varphi_p^{\text{est}}(t)] = -\frac{1}{3!(2\pi)^2}\int_{\mathbb{R}^2} d\vec{\omega}\, G_p(\vec{\omega}, t)\, S_2(\vec{\omega}) + \Theta[t^5], \quad (25)$$

$$\text{var}[\varphi_p^{\text{est}}(t)] = \left(\frac{\mu_y}{\mu_x^2 + \mu_y^2}\right)^2 \text{var}[\sigma_x^{\text{est}}] + \left(\frac{\mu_x}{\mu_x^2 + \mu_y^2}\right)^2 \text{var}[\sigma_y^{\text{est}}] + F_p(0,t)^2\, \text{var}[\mu_B^{\text{est}}]. \quad (26)$$

Note that $\mathbb{E}[\varphi_p^{\text{est}}(t)]$ depends to leading order on the bispectrum, unlike $\mathbb{E}[\phi_p^{\text{est}}(t)]$ above.

8.2 Restriction to the principal domain

For any real, classical process, the bispectrum has three general symmetries: (1) $S_2(\omega_1, \omega_2) = S_2(\omega_2, \omega_1)$ (permutation symmetry); (2) $S_2(\omega_1, \omega_2) = S_2(-\omega_1, -\omega_2)$ (invariance under complex conjugation); (3) $S_2(\omega_1, \omega_2) = S_2(-\omega_1 - \omega_2, \omega_2)$ (stationarity). These symmetries define the 12 regions of the frequency plane depicted in Fig. 3c. If $(\omega_1, \omega_2) \in \text{int}(\mathcal{D}_2)$ is contained in the interior of the principal domain, the symmetries imply

$$S_2(\omega_1, \omega_2) = S_2(\omega_2, \omega_1) = S_2(-\omega_2, \omega_1 + \omega_2) = S_2(-\omega_1, \omega_1 + \omega_2) = S_2(-\omega_1 - \omega_2, \omega_1) = S_2(-\omega_1 - \omega_2, \omega_2) = S_2(-\omega_1, -\omega_2) = S_2(-\omega_2, -\omega_1) = S_2(\omega_2, -\omega_1 - \omega_2) = S_2(\omega_1, -\omega_1 - \omega_2) = S_2(\omega_1 + \omega_2, -\omega_1) = S_2(\omega_1 + \omega_2, -\omega_2).$$

In other words, the bispectrum takes a value equivalent to $S_2(\omega_1, \omega_2)$ in each of the 12 regions. This is summarized by the multiplicity, $m(\omega_1, \omega_2) = 12$. For (ω, ω), which lies on the boundary of $\mathcal{D}_2$,

$$S_2(\omega, \omega) = S_2(-\omega, -\omega) = S_2(-2\omega, \omega) = S_2(\omega, -2\omega) = S_2(2\omega, -\omega) = S_2(-\omega, 2\omega),$$

implying $m(\omega, \omega) = 6$. For $(\omega, 0)$, which also lies on the boundary of $\mathcal{D}_2$,

$$S_2(\omega, 0) = S_2(0, \omega) = S_2(-\omega, 0) = S_2(0, -\omega) = S_2(\omega, -\omega) = S_2(\omega, -\omega),$$

similarly implying $m(\omega, 0) = 6$. Note that $(\omega_1, \omega_2) = (0, 0)$ is invariant under all of the symmetries, implying that $m(0, 0) = 1$.

The symmetries and multiplicities simplify the expected value of the phase substantially. Recall that after $M \gg 1$ repetitions of control sequence p with cycle time T, the frequency comb approximation enables us to write the expected phase as a discrete sum depending on the bispectrum and the FF evaluated at the harmonic frequencies,

$$\mathbb{E}[\varphi_p^{\text{est}}(MT)] = -\frac{1}{3!(2\pi)^2} \int_{\mathbb{R}^2} d\vec{\omega}\, G_p(\vec{\omega}, MT) S_2(\vec{\omega}) \approx -\frac{M}{3!T^2} \sum_{\vec{k} \in \mathbb{Z}^2} G_p(\omega_h \vec{k}, T) S_2(\omega_h \vec{k}).$$

In terms of the multiplicities, we can rewrite the sum as

$$\begin{aligned}\mathbb{E}[\varphi_p^{\text{est}}(MT)] \approx &-\frac{12M}{3!T^2} \sum_{\omega_h \vec{k} \in \text{int}(\mathcal{D}_2)} G_p(\omega_h \vec{k}, T) S_2(\omega_h \vec{k}) - \frac{6M}{3!T^2} \sum_{k \in \mathbb{Z}} G_p(\omega_h k, \omega_h k, T) S_2(\omega_h k, \omega_h k) \\ &- \frac{6M}{3!T^2} \sum_{k \in \mathbb{Z}} G_p(\omega_h k, 0, T) S_2(\omega_h k, 0) - \frac{M}{3!T^2} G_p(0, 0, T) S_2(0, 0) \\ = &-\frac{M}{3!T^2} \sum_{\omega_h \vec{k} \in \mathcal{D}_2} m(\omega_h \vec{k}) G_p(\omega_h \vec{k}, T) S_2(\omega_h \vec{k}).\end{aligned}$$

Using $S_2(\omega_h \vec{k}) = S_2(-\omega_h \vec{k})$ and $G_p(\omega_h \vec{k}, T)^* = G_p(-\omega_h \vec{k}, T)$, and truncating the sum to a finite subset $\mathcal{K}_2$, we obtain

$$\mathbb{E}[\varphi_p^{\text{est}}(MT)] \approx -\frac{M}{3!T^2} \sum_{\vec{k} \in \mathcal{K}_2} m(\omega_h \vec{k}) \text{Re}[G_p(\omega_h \vec{k}, T)] S_2(\omega_h \vec{k}). \tag{27}$$

8.3 Likelihood function $P(\vec{\varphi}|\vec{S}_2)$

Given the actual bispectrum in $\mathcal{K}$, $\vec{S}_2 = [S_2(\omega_h \vec{k}_1), \dots, S_2(\omega_h \vec{k}_N)]^T$, the conditional probability of measuring $\varphi_p^{\text{est}}(MT)$ follows from Eqs. (25)-(27),

$$P\big[\varphi_p^{\text{est}}(MT)|\vec{S}_2\big] = \frac{1}{\sqrt{2\pi\, \text{var}[\varphi_p^{\text{est}}(MT)]}} \exp\left\{ -\frac{\Big[\varphi_p^{\text{est}}(MT) + \frac{M}{3!T^2} \sum_{\vec{k} \in \mathcal{K}} m(\omega_h \vec{k}) \text{Re}[G_p(\omega_h \vec{k}, T)] S_2(\omega_h \vec{k})\Big]^2}{2\, \text{var}[\varphi_p^{\text{est}}(MT)]} \right\}. \tag{28}$$

Reconstructing the bispectrum requires measurements the non-Gaussian phase for a set of control sequences $p = 1, \dots, P$ with $P \geq N$, which we gather into the column vector $\vec{\varphi} = [\varphi_1^{\text{est}}(MT), \dots, \varphi_P^{\text{est}}(MT)]^T$. Because the non-Gaussian phase measurements are uncorrelated, the likelihood or probability of obtaining $\vec{\varphi}$ given $\vec{S}_2$ is a product of the conditional probabilities for the complete set of control sequences,

$$P(\vec{\varphi}|\vec{S}_2) = \prod_{p=1}^{P} P\big[\varphi_p^{\text{est}}(MT)|\vec{S}_2\big] = \big[(2\pi)^P \det \mathbf{\Sigma}\big]^{-\frac{1}{2}} \exp\left[-\frac{1}{2}(\vec{\varphi} - \mathbf{A}\vec{S}_2)^T \mathbf{\Sigma}^{-1} (\vec{\varphi} - \mathbf{A}\vec{S}_2)\right]. \tag{29}$$

Here, the $P \times P$ covariance matrix $\mathbf{\Sigma}$ is diagonal with elements $\mathbf{\Sigma}_{p,q} = \text{var}[\varphi_p^{\text{est}}(MT)]\, \delta_{p,q}$, and the $P \times N$ reconstruction matrix $\mathbf{A}$ depends on the filter functions evaluated at the harmonic frequencies,

$$(\mathbf{A})_{p,n} = -\frac{M}{3!T^2}\, m(\omega_h \vec{k}_n)\, \text{Re}[G_p(\omega_h \vec{k}_n, T)]. \tag{30}$$

In the experiment, the likelihood in Eq. (29) is determined by measuring the non-Gaussian phase for each of the $P = 11$ control sequences in Fig. (3). Since we also rely on these sequences to estimate the PSD, both the bispectrum and the PSD can be estimated with the the same set of transverse Pauli measurements. For each of the control sequences, $\varphi_p^{\text{est}}(MT)$ was determined from Eq. (22) using measurements of σ_x^{est}, σ_y^{est} and μ_B^{est}. The variances of the $\varphi_p^{\text{est}}(MT)$, which constitute the covariance matrix $\mathbf{\Sigma}$, are given by Eq. (26) with μ_x and μ_y replaced by the estimated values σ_x^{est}, σ_y^{est}. The reconstruction matrix $\mathbf{A}$ is determined from Eq. (30), with each FF evaluated on the set of harmonics $\mathcal{K}_1$ depicted in Fig. 3(b).

8.4 Regularized maximum likelihood estimation

For the Gaussian likelihood derived in the previous section, the maximum likelihood estimate (MLE) of the bispectrum is equivalent to the value of $\vec{S}_2$ that minimizes the exponent in Eq. (29),

$$\vec{S}_2^{\mathrm{MLE}} = \underset{\vec{S}_2}{\operatorname{argmin}} \frac{1}{2}\left(\vec{\varphi} - \mathbf{A}\vec{S}_2\right)^T \mathbf{\Sigma}^{-1}\left(\vec{\varphi} - \mathbf{A}\vec{S}_2\right). \tag{31}$$

In the special case where $\mathbf{\Sigma} \propto \mathbf{I}$, we recover the least-squares estimate used in Ref. [4] with solution $\vec{S}_2^{\mathrm{LS}} = \mathbf{A}^{-1}\vec{\varphi}$. Even with a nonuniform covariance matrix, Eq. (31) is a simple convex optimization problem admitting an analytic solution for $\vec{S}_2^{\mathrm{MLE}}$. When $\mathbf{A}$ is ill-conditioned, however, the MLE suffers from numerical instability, which can introduce significant error into the estimate of the bispectrum, despite the existence of an analytic solution. The problem can be made more stable by introducing a regularization term or "regularizer" $Q(\vec{S}_2)$, producing the regularized maximum likelihood estimate (RMLE) of the bispectrum,

$$\vec{S}_2^{\mathrm{RMLE}} = \underset{\vec{S}_2}{\operatorname{argmin}} \left[\frac{1}{2}(\mathbf{A}\vec{S}_2 - \vec{\varphi})^T\mathbf{\Sigma}^{-1}(\mathbf{A}\vec{S}_2 - \vec{\varphi}) + Q(\vec{S}_2)\right]. \tag{32}$$

The regularizer imposes additional structure on the solution, making it more robust to numerical instability arising from $\mathbf{A}$. It also prevents overfitting, in which the estimated bispectrum is unduly influenced by errors in $\vec{\varphi}$ and is, thus, a poor predictor of the qubit dynamics under more general control settings.

There are numerous methods of regularization for ill-conditioned and/or ill-posed problems. An approach particularly ammenable to maximum likelihood estimation is Tikhonov regularization, which employs an L2 regularizer $Q(\vec{S}_2) = \|\lambda\vec{S}_2\|_2^2$ with strength controlled by the regularization parameter $\lambda \geq 0$ [S5]. In Eq. (32), this regularizer has the effect of penalizing $\vec{S}_2$ with larger L_2-norms. To estimate the bispectrum, we consider a variation of Tikhonov regularization in which

$$Q(\vec{S}_2) = \left\|\lambda\mathbf{D}\vec{S}_2\right\|_2^2, \tag{33}$$

where $\mathbf{D} = \mathrm{diag}\,(d_1, \cdots, d_N)$ is the diagonal "smoothing matrix". Note that the Tikhonov regularizer is recovered when $d_1 = \ldots = d_N = 1$ (Fig. 5a) and the standard maximum likelihood estimate is recovered when $\lambda = 0$. Using non-uniform values for the diagonals enables us to incorporate prior information about the bispectrum. For example, if the magnitude of the bispectrum is known to decay at the high-frequency border of $\mathcal{K}$, we can make the corresponding harmonics in $\mathbf{D}$ large compared to those of the interior (Fig. 5b). Such a smoothing matrix favors a solution with small magnitude at the border. The connection between the smoothing matrix and prior knowledge of the bispectrum is more explicit in a Bayesian formulation of the estimation problem in which the RMLE estimate in Eq. (32) with the regularizer in Eq. (33) is equivalent to a posterior mean estimate in which the prior distribution of the bispectrum is Gaussian and zero-mean with covariance matrix $(2\lambda^2\mathbf{D}^2)^{-1}$, provided $\mathbf{D}$ is full-rank. For any smoothing matrix, the regularized maximum likelihood estimate has the simple analytic solution,

$$\vec{S}_2^{\mathrm{RMLE}} = \left(\mathbf{A}^T\mathbf{\Sigma}^{-1}\mathbf{A} + 2\lambda^2\mathbf{D}^2\right)^{-1}\left(\mathbf{A}^T\mathbf{\Sigma}^{-1}\vec{\varphi}\right). \tag{34}$$

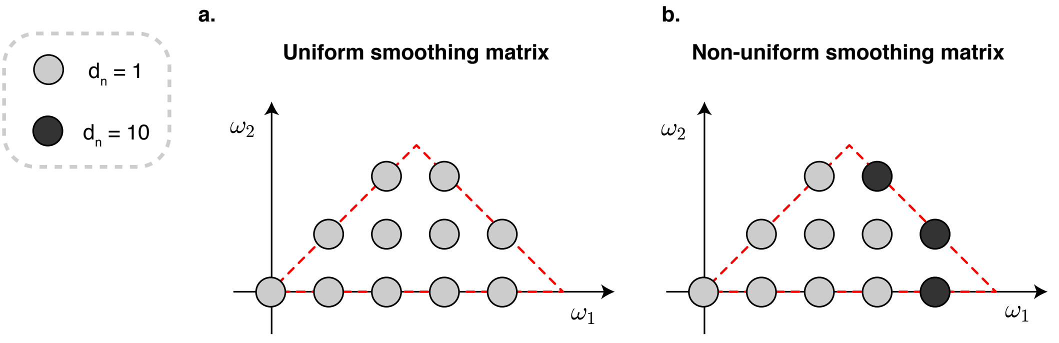

Supplementary Figure 5. Smoothing matrix $\mathbf{D}$ given a prior information. a, A smoothing matrix that assumes the least prior information about the bispectrum. **b,** A smoothing matrix that assumes the bispectrum decays to zero at the border of an octant.

8.5 The L-curve criterion

Although it guards against numerical instability and overfitting to errors in the measured data, the regularizer can introduce its own error into the estimate if λ too large. A fundamental challenge in regularization is selecting a value of λ that balances these sources of error. While this problem is still an active area of research, one of the most widely used strategies for selecting λ is the L-curve criterion [S6]. A graphical technique, the L-curve criterion enables one to visualize the magnitude of the regularization error in proportion to other errors in the estimate and choose λ accordingly.

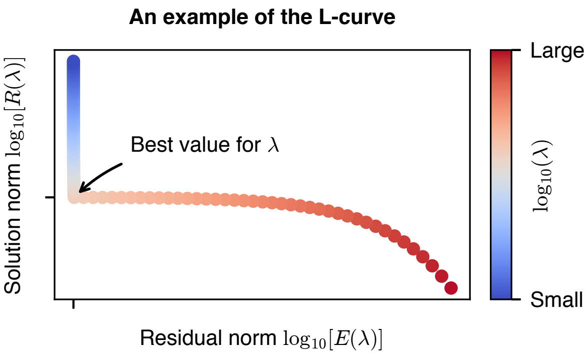

Supplementary Figure 6. An example of the L-curve plot.

For a given λ, the sources of error that contribute to the regularized maximum likelihood estimate in Eq. (34) are described by the residual norm

$$E(\lambda) \equiv \left[\frac{1}{2}(\mathbf{A}\vec{S}_2^{\text{RMLE}} - \vec{\varphi})^T\, \mathbf{\Sigma}^{-1}(\mathbf{A}\vec{S}_2^{\text{RMLE}} - \vec{\varphi})\right]^{1/2}. \tag{35}$$

and the solution norm

$$R(\lambda) \equiv \left\|\mathbf{D}\vec{S}_2^{\text{RMLE}}\right\|_2 = \left\|\text{diag}(d_1, \cdots, d_{|\mathcal{R}|})\vec{S}_2^{\text{RMLE}}\right\|_2. \tag{36}$$

The regularization parameter λ enters both the residual norm and solution norm implicitly through $\vec{S}_2^{\text{RMLE}}$. The residual norm increases as λ grows, while the solution norm decreases. When $E(\lambda)$ is too large relative to $R(\lambda)$, the estimate does not account for the measured data due to error introduced by the regularization. Conversely, when $E(\lambda)$ is too small relative to $R(\lambda)$, the estimate is fits the measured data too closely, making it susceptible to overfitting and numerical error. The influence of λ on the error conributions is captured by the L-curve, a parametric plot of $\log R(\lambda)$ vs. $\log E(\lambda)$ as a function of λ. A typical L-curve, with its characteristic "L" shape, is shown in Fig. 6. Note that as λ increases from left to right, $\log R(\lambda)$ sharply decreases and then plateaus, while $\log E(\lambda)$ is initially stable followed by a rapid increase. The corner of the L-curve, marks a point at which the solution norm and residual norm are small simultaneously. The corner, thus, signifies the optimal value of λ according to the L-curve criterion.

Figure 7 shows L-curves generated by our experimental data for the two different smoothing matrices illustrated in Fig. 5. For both the uniform and non-uniform smoothing matrices, the L-curves lack corners. Unlike the typical L-curve in Fig. 6, $\log R(\lambda)$ does not exhibit a sharp increase as $\lambda \to 0$. This indicates that, for the control sequences we have selected, the reconstruction matrix $\mathbf{A}$ is sufficiently well conditioned to make regularization the dominant source of error [S7]. Consequently, it is not optimal to utilize regularization in this setting and the reconstruction presented in the main text uses $\lambda = 0$.

Note that this finding is contingent on both $\mathbf{A}$ and the particular regularizer we employ. Estimating the bispectrum at a greater number of harmonics demands a larger $\mathbf{A}$, which is more likely to be near singular and/or poorly conditioned. This scenario will likely require some form of regularization. Additionally, the error introduced by regularization is reduced when prior knowledge of the bispectrum (if available) is used to select the regularizer. For example, suppose a noise model or previous experiment indicates that $\vec{S}_2$ takes a value in the vicinity of $\vec{S}_\mu$. This information is captured by the regularizer

$$Q(\vec{S}_2) = \frac{1}{2}(\vec{S}_2 - \vec{S}_\mu)^T(2\lambda^2\mathbf{D}^2)(\vec{S}_2 - \vec{S}_\mu). \tag{37}$$

This corresponds to a Gaussian prior distribution of $\vec{S}_2$ with mean $\vec{S}_\mu$ and covariance matrix $(2\lambda^2\mathbf{D}^2)^{-1}$. In contrast, naively employing Tikhonov regularization amounts to assuming a zero-mean prior distribution of $\vec{S}_2$.

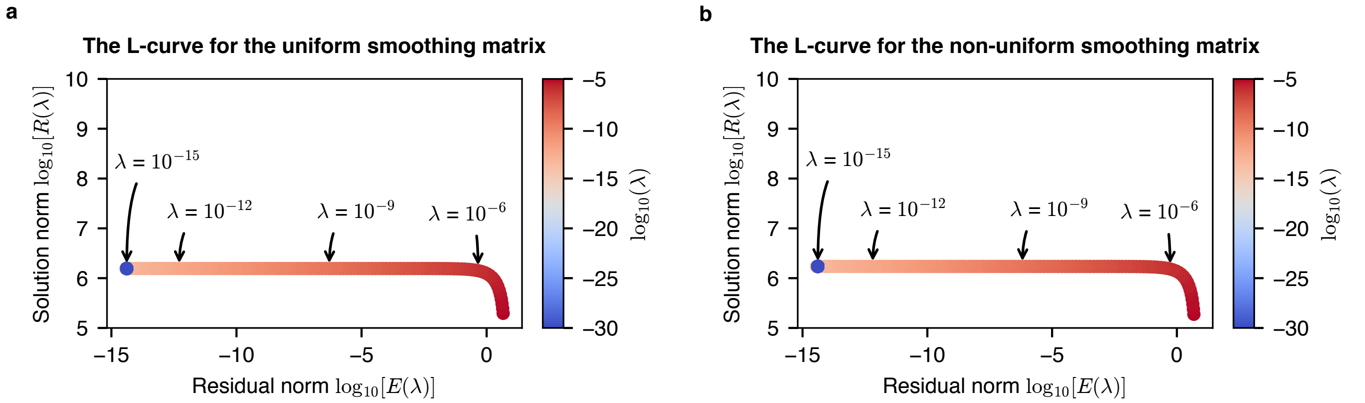

Supplementary Figure 7. The L-curve plots for experimental data. a, The L-curve plot for the uniform smoothing matrix. **b,** The L-curve plot for the non-uniform smoothing matrix.

Supplementary References

S1. V. E. Manucharyan, J. Koch, L. I. Glazman, and M. H. Devoret, "Fluxonium: Single Cooper-pair circuit free of charge offsets," Science **326**, 113–116 (2009).

S2. E. Magesan, J. M. Gambetta, and J. Emerson, "Scalable and robust randomized benchmarking of quantum processes," Phys. Rev. Lett. **106**, 180504 (2011).

S3. C. Macklin, K. O'Brien, D. Hover, M. E. Schwartz, V. Bolkhovsky, X. Zhang, W. D. Oliver, and I. Siddiqi, "A near-quantum-limited Josephson traveling-wave parametric amplifier," Science **350**, 307–310 (2015).

S4. G. Casella and R. L. Berger, Statistical Inference (Duxbury Pacific Grove, CA, 2002).

S5. A. Tikhonov, "Solution of incorrectly formulated problems and the regularization method," Dokl. Akad. Nauk **151**, 1035–1038 (1963).

S6. P. C. Hansen, "The L-curve and its use in the numerical treatment of inverse problems," in *Computational Inverse Problems in Electrocardiology* (WIT Press, 2000) pp. 119–142.

S7. T. Regińska, "A regularization parameter in discrete ill- posed problems," SIAM J. Sci. Comput. **17**, 740–749 (1996).
